# Rescue treatment with terlipressin in children with refractory septic shock: a clinical study

**DOI:** 10.1186/cc3984

**Published:** 2006-01-31

**Authors:** Antonio Rodríguez-Núñez, Jesús López-Herce, Javier Gil-Antón, Arturo Hernández, Corsino Rey, the RETSPED Working Group of the Spanish Society of Pediatric Intensive Care

**Affiliations:** 1 Clinical Assistant, Pediatric Emergency and Critical Care Division, Department of Pediatrics, Hospital Clínico Universitario de Santiago de Compostela, Servicio Galego de Saude (SERGAS) and University of Santiago de Compostela, Santiago de Compostela, Spain; 2 Clinical Assistant, Pediatric Intensive Care Unit, Hospital General Universitario Gregorio Marañón, Madrid, Spain; 3 Clinical Assistant, Pediatric Intensive Care Unit, Hospital de Cruces, Barakaldo, Spain; 4 Clinical Assistant, Pediatric Intensive Care Unit, Hospital Puerta del Mar, Cádiz, Spain; 5 Director, Pediatric Intensive Care Unit, Hospital Universitario Central de Asturias, Oviedo, Spain

## Abstract

**Introduction:**

Refractory septic shock has dismal prognosis despite aggressive therapy. The purpose of the present study is to report the effects of terlipressin (TP) as a rescue treatment in children with catecholamine refractory hypotensive septic shock.

**Methods:**

We prospectively registered the children with severe septic shock and hypotension resistant to standard intensive care, including a high dose of catecholamines, who received compassionate therapy with TP in nine pediatric intensive care units in Spain, over a 12-month period. The TP dose was 0.02 mg/kg every four hours.

**Results:**

Sixteen children (age range, 1 month–13 years) were included. The cause of sepsis was meningococcal in eight cases, *Staphylococcus aureus *in two cases, and unknown in six cases. At inclusion the median (range) Pediatric Logistic Organ Dysfunction score was 23.5 (12–52) and the median (range) Pediatric Risk of Mortality score was 24.5 (16–43). All children had been treated with a combination of at least two catecholamines at high dose rates. TP treatment induced a rapid and sustained improvement in the mean arterial blood pressure that allowed reduction of the catecholamine infusion rate after one hour in 14 out of 16 patients. The mean (range) arterial blood pressure 30 minutes after TP administration increased from 50.5 (37–93) to 77 (42–100) mmHg (*P *< 0.05). The noradrenaline infusion rate 24 hours after TP treatment decreased from 2 (1–4) to 1 (0–2.5) µg/kg/min (*P *< 0.05). Seven patients survived to the sepsis episode. The causes of death were refractory shock in three cases, withdrawal of therapy in two cases, refractory arrhythmia in three cases, and multiorgan failure in one case. Four of the survivors had sequelae: major amputations (lower limbs and hands) in one case, minor amputations (finger) in two cases, and minor neurological deficit in one case.

**Conclusion:**

TP is an effective vasopressor agent that could be an alternative or complementary therapy in children with refractory vasodilatory septic shock. The addition of TP to high doses of catecholamines, however, can induce excessive vasoconstriction. Additional studies are needed to define the safety profile and the clinical effectiveness of TP in children with septic shock.

## Introduction

Septic shock is a severe clinical condition with a complex pathophysiology and poor prognosis despite intensive therapy [1,2]. In sepsis, a cascade of macrocirculatory and microcirculatory alterations may induce an inability to maintain vasoconstriction, and can lead to severe hypotension [3]. When hypotension becomes refractory to current intensive treatments, the prognosis of septic shock is very poor [4,5].

Prompted by the desperate situation of patients who fail to respond to aggressive therapy with fluid expansion, vasopressors, inotropes, and other therapies, alternative or complementary vasoconstrictors have been used [3]. Vasopressin (AVP) has potent vasoconstrictive effects mediated via V_1 _receptors and has been shown effective in catecholamine-resistant hypotension due to septic shock [5-10].

Terlipressin (TP) is a synthetic analog of AVP with a similar pharmacodynamic profile, but with a significantly longer half-life, that has showed promising effects in some case reports of adult patients [11-16] and of children with refractory vasodilatory septic shock [4,17-19]. On the other hand, concerns have been raised about possible adverse effects of these alternative pressor agents [20-22]. New clinical evidence is therefore needed to define the role of both AVP and TP in vasodilatory septic shock [4,15,22,23].

In the present article, we report the results of the use of TP as a last-resource compassionate therapy in critically ill children with catecholamine-resistant hypotension due to septic shock.

## Patients and methods

A prospective, multicenter, observational study was carried out in nine pediatric intensive care units (PICUs) in Spain, during a 12-month period (July 2004–June 2005). Indication of treatment was made by the responsible physician, and administrative authorization was obtained after fulfillment of the strict legal and ethical conditions for compassionate use of drugs required in our country [24]. Briefly, compassionate therapy permits the use of a non-licensed drug or a drug licensed for other indications, outside a clinical trial, in desperate clinical situations where the responsible doctor considers that no other therapeutic alternatives exist and after a specific informed consent process has been carried out.

Inclusion criteria included septic shock with refractory hypotension, defined by an inability to maintain a mean arterial pressure (MAP) above the third percentile for age despite fluid resuscitation and 'high catecholamine doses' (at least 1 µg/kg/min noradrenaline or adrenaline, associated with variable doses of dopamine and/or dobutamine), or evidence of adverse effects of catecholamines (ischemia, arrhythmias). Patients aged from one month to 15 years were eligible. Children with cardiac diseases were excluded.

Due to the lack of specific treatment recommendations, we decided to maintain the TP dosage used in previous pediatric cases [17]: 0.02 mg/kg every four hours by intravenous bolus for a maximum of 72 hours. The main objective of TP treatment was to improve survival of the episode; specific objectives were to achieve and maintain MAP values within the normal range for age and, when possible, to lessen the noradrenaline and adrenaline infusion rates.

### Statistical analysis

Values are presented as the median (range). Nonparametric tests were used and intragroup comparisons were performed using the Wilcoxon test. *P *< 0.05 was considered statistically significant. The inotropic equivalent was calculated by means of a previously described formula [25].

## Results

Sixteen children, with ages ranging from one month to 13 years, were included in the study. Patient characteristics are presented in Table 1. Sepsis was caused by *Neisseria meningitides *in eight cases and by *Staphylococcus aureus *in two cases; no bacteria were isolated in the remaining six children (sepsis was of nosocomial origin in three cases). At PICU admission, the median (range) Pediatric Logistic Organ Dysfunction score was 23.5 (12–52) and the median (range) Pediatric Risk of Mortality score was 24.5 (16–43). Seven patients already had signs of ischemia at the time TP treatment was considered (Table 1). Six patients had acute renal failure, four patients had coagulopathy, three patients had severe acidosis, two patients had rhabdomyolysis, two patients had acute respiratory distress syndrome, and one patient had refractory intracranial hypertension. Two children had been resuscitated from cardiac arrest (Table 1).

Prior to the start of TP treatment, 15 patients were being mechanically ventilated and ten patients were being treated with continuous renal replacement therapy. Corticosteroids were administered to eight children, and other treatments (antithrombin III, treatment of intracranial hypertension, plasmapheresis, fresh frozen plasma and activated C protein) were each used in one case, respectively. All patients received a combination of at least two catecholamines at high doses. The median (range) rates were 21.5 (10–52) µg/kg/min for dopamine (16 patients), 22.5 (5–40) µg/kg/min for dobutamine (12 patients), 2 (1–4) µg/kg/min for noradrenaline (14 patients), and 1.25 (0.4–4) µg/kg/min for adrenaline (12 patients). Three children also received milrinone, and another child also received digoxine.

TP was started 24 (4–168) hours after admission and was maintained for 24 (3–102) hours (Table 2). The hemodynamic variables and catecholamine infusion rates after TP therapy are summarized in Table 3.

The MAP significantly increased in all patients after TP administration, from 50.5 (37–93) mmHg pre TP administration, to 77 (42–100) mmHg 30 minutes after TP administration, and to 69.5 (41–104) mmHg 1 hour after TP administration (*P *< 0.05). The heart rate did not change significantly (Table 3).

Treatment with TP permitted a significant reduction in the noradrenaline infusion rate, from 2 (1–4) µg/kg/min pre TP administration, to 1 (0–2.6) µg/kg/min 12 hours after TP administration, and to 1 (0–2.5) µg/kg/min 24 hours later (*P *< 0.05) (Table 3).

**Figure 1:**
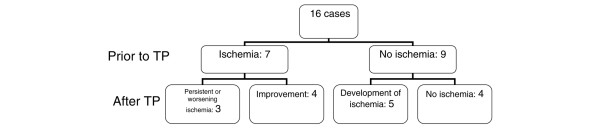
Evolution of limbs and/or cutaneous ischemia after terlipressin (TP) treatment.

Seven patients showed signs of ischemia prior to TP administration; ischemia persisted or increased with TP treatment in three cases, and improved in four cases (Figure 1). The other nine patients had no signs of ischemia before TP therapy was started. In this subset of nine patients, five developed ischemia possibly related to TP treatment (Figure 1), one of which showed severe limb and intestinal ischemia.

The responsible physicians considered that TP treatment could be also related to other adverse effects: oliguria in two cases, rhabdomyolysis in two cases, hyperkalemia in one case, and hyperbilirrubinemia in another child (Table 2).

Seven patients survived the septic shock episode and nine children died. Causes of death were refractory shock in three cases, refractory arrhythmia in three cases, withdrawal of therapy in two cases, and multiorgan failure in one case (Table 2).

In an adolescent with severe cranial trauma and refractory intracranial hypertension, who developed a nosocomial sepsis with severe hypotension and acute renal failure, TP administration produced severe cutaneous and limb ischemia that was considered by the attending physician a direct factor contributing to death. One infant survived the shock episode but died two weeks later, due to intractable propionic acidemia.

 In our patients, the Pediatric Risk of Mortality score or the Pediatric Logistic Organ Dysfunction score, age, sex, the time elapsed until the start of TP administration, the catecholamine infusion rate, the MAP, treatments with steroids, or the length of stay in the PICU were not associated with mortality. Four of the survivors developed sequelae. One patient suffered a major limb amputation, including both lower limbs (below knees) and both hands. Two children suffered the amputation of one finger, and another patient developed dysmetria and partial anopsy. The length of the PICU stay was 8 (1–51) days (Table 2).

## Discussion

Septic shock is a very complex condition, characterized by circulatory failure. Its treatment has been based, in addition to antibiotic therapy, on aggressive volume resuscitation and cardiocirculatory support by means of the vasopressor and inotropic effects of catecholamines [1-3,15,26,27]. Despite this approach and intensive care and monitoring, septic shock mortality and morbidity remain very high. New therapies are therefore urgently needed [26,27].

AVP plasma concentrations are very high in cardiogenic or hypovolemic shock [1,28]. In septic shock, however, a biphasic response has been recognized, with high levels in the early phase and inappropriately low AVP levels in established septic shock [1,28,29]. This evidence and the potent vasopressor effects of AVP prompted its use in vasodilatory septic shock. AVP has been effective in restoring the MAP and vascular tone in adult patients [5-9,26] as well as in some pediatric case series [10,30]. AVP has been also beneficial in the treatment of excessive vasodilation associated with cardiopulmonary bypass [31] and in postcardiotomy shock resistant to catecholamine therapy [32-34].

TP is a long-acting synthetic analog of AVP that has also demonstrated significant vasopressor effects in animal models [35,36], in adult patients with norepinephrine-resistant septic shock [11,13,14,16], and in a few pediatric cases with vasodilatory shock [4,17-19]. A recently published trial comparing the short-time effects (only six hours) of noradrenaline and TP treatment in adult patients with hyperdynamic septic shock indicates that both drugs are effective in raising the MAP and improving renal function [16].

To our knowledge, results of randomized clinical trials to ascertain the effects of TP treatment, alone or in combination with noradrenaline or other catecholamines, in pediatric vasodilatory septic shock are lacking. The few case reports available [4,17-19], however, suggest that TP has a possible role in intractable septic shock, an issue that should be explored.

We had previously reported the use of TP in four children with septic shock resistant to high doses of noradrenaline, combined with other catecholamines. In these patients TP therapy induced a rapid and sustained improvement in MAP, which allowed the lessening or even withdrawal of noradrenaline infusion, without related adverse effects. Two patients survived [17].

Matok and colleagues recently reported their retrospective experience with TP therapy in 14 children who suffered 16 septic shock episodes [4]. They observed significant improvements in respiratory and hemodynamic indices shortly after TP treatment. Adrenaline infusion was decreased or stopped in eight patients. Six patients survived. No reference to adverse effects was reported in this group of patients. Although all of the children were considered to be in an extreme state of septic shock, eight patients had undergone correction of congenital heart disease so a component of cardiogenic shock cannot be ruled out, and this fact could interfere with the interpretation of results.

The present study is the first prospective and observational study to report the clinical effects of TP administered as compassionate therapy in children with refractory hypotension due to septic shock. The patients were followed up until death or PICU discharge. To avoid bias, we have excluded patients with cardiac diseases. One-half of the patients had meningococcal purpura fulminans. Despite these differences in the patient characteristics analyzed, our results are comparable with those of Matok and colleagues [4]. We have also observed a significant increase in the MAP that permitted decreasing the noradrenaline infusion rate without changes in the heart rate (Table 3).

Noradrenaline and adrenaline, particularly at high doses, have potent vasoconstrictive effects that can lead to irreversible tissue ischemia [2,26,27]. Similar concerns arise when AVP and TP are considered for reversing severe vasodilation in septic shock [15,21,23]. When TP is used as a last-resource compassionate therapy, as in the present study, it is added to the previous treatment, which in this case included combinations of catecholamines in high doses. Such a synergy of effects with an increase of previous tissue perfusion insufficiency or its development could therefore be anticipated. In our series, seven patients had signs of ischemia before TP administration; interestingly, while ischemia persisted in three of them, it improved in four children (Figure 1). On the other hand, five out of nine patients without signs of ischemia developed skin and/or limb ischemia after adding TP to the catecholamine dose (Figure 1).

This heterogeneous response is intriguing. We can speculate that improvement of tissue perfusion improvement could be an indirect effect of restoring the MAP and that the development or worsening of ischemia could result from the addition of vasoconstrictive effects of catecholamines and TP or from a direct effect of TP administration. It also appears from our results that TP requirements may have great variability derived from multiple patient characteristics, and the dosage should be titrated according to clinical consequences (balance between positive and adverse effects). One potential strategy in this sense could be to administer a loading dose of TP followed by a goal-directed variable intravenous infusion rate [37].

Another point to be elucidated is the most adequate bolus dose of TP. Due to the lack of specific dosage recommendations, we decided to use the same dosage as that utilized in our previous study [17]: intermittent intravenous doses of 0.02 mg/kg every 4 hours for a maximum of 72 hours. This dose was based on arbitrary extrapolations from doses used for other indications in adults [38,39] and it was considered a 'low dose'; nonetheless, a subset of patients developed ischemia. Further studies are therefore needed to ascertain the ideal dosage and schedule in children with vasodilatory septic shock. In this sense, a clinical tool to monitor vasoconstriction at tissue level could be very useful. Some case reports have been published in which gastric tonometry [20,40], the ileal pCO_2 _gap [41], and the sublingual microcirculatory flow [42] have been used to monitor splanchnic and sublingual microvasculature after treatment with AVP or TP.

Our results indicate that TP may have a role in the therapy of refractory hypotension. TP administration might have influenced the final prognosis of our patients. Moreover, consider that nearly all of our patients had a desperate clinical situation and were treated with TP as a last resource. In our opinion, at least three children were treated in a near-death situation. In this condition, seven of 16 children survived the septic shock episode – a figure similar to that reported by Matok and colleagues [4]. On the other hand, in one case the attending physicians considered that TP was a major factor of the bad outcome, and in two patients they decided on withdrawal of therapy due to severe ischemia, multiple organ failure, and anticipation of non-acceptable sequelae. Only one of seven survivors had severe ischemic sequelae, with amputation of lower limbs below the knees and both hands; another two children suffered the amputation of one finger.

Our study has several limitations. Compassionate use of drugs permits the administration of nonproven therapies, outside clinical trials, in desperate cases; due to this fact, however, the treatment has a high risk of being a delayed, and therefore futile, treatment. It can be argued that if there is a rational indication for the treatment in the light of available evidence, then to have some chance of success TP therapy should be started before the clinical situation becomes so deteriorated that treatment is worthless.

Another drawback is the small number of patients included and the fact that they were gathered from nine different hospitals. This is justified by the fact that vasodilatory septic shock refractory to catecholamines is rare in children [2] and therefore multicenter studies are required. The number of cases precludes statistical analysis to detect factors that are correlated with clinical response, adverse effects, and prognosis.

Also, in order to evaluate the effects of TP administration in further detail, certain additional hemodynamic data, such as systemic vascular resistance, the cardiac index, or calorimetry measurements, could have been very useful. These data were unfortunately not available in most of our patients.

## Conclusion

TP therapy is effective for reversing hypotension in children with catecholamine-resistant septic shock. This treatment may cause significant ischemic injury and it should be considered a last-resource treatment in the critical care setting. Our results nevertheless indicate that TP is a promising treatment, and they give support for future controlled clinical trials to assess the efficacy, safety, dosage, and indications of TP in pediatric vasodilatory septic shock.

## Key messages

• 	TP therapy is effective for reversing hypotension in children with catecholamine-resistant septic shock.

• 	TP therapy permitted lowering of the high doses of noradrenaline needed in these patients.

• 	TP therapy in combination with catecholamine vasopressors may cause significant ischemic injury.

• 	Controlled clinical trials are needed to assess the efficacy, safety, dosage, and indications of TP therapy in pediatric vasodilatory septic shock.

## Abbreviations

AVP = vasopressin; MAP = mean arterial pressure; PICU = pediatric intensive care unit; TP = terlipressin.

## Competing interests

The authors declare that they have no competing interests. This study was partially supported by Ferring, S.A., Madrid, Spain (organization of two working meetings).

## Authors' contributions

ARN conceived, designed, and coordinated the study, reviewed all necessary material, performed statistical analysis, and wrote the initial and successive drafts. JLH participated in the design of the study and critically reviewed the drafts. JGA and AH critically reviewed the drafts of the manuscript. CR assisted with study design and assessment of manuscript. ARN, JLH, JGA, AH, CR, and other members of the RETSPED Working Group of the Spanish Society of Pediatric Intensive Care (VM, CPC, ASG, JDLC, MTH, AM, and FMT) participated in the working meetings, discussed the design of the study, were in charge of the reported patients, and fulfilled the case records. All authors gave final approval of the version to be published.

## Figures and Tables

**Table 1:** Clinical characteristics of patients before terlipressin treatment

Patient	Sex	Age (months)	Weight (kg)	Underlying disease	Cause of sepsis	Pediatric Logistic Organ Dysfunction score	Pediatric Risk of Mortality score	Prior ischemia	Other data
1	Male	15	12	No	Meningococcus	34	38	Limbs, cutaneous	
2	Female	156	47	No	Meningococcus	22	17	No	Coagulopathy
3	Female	36	15	VATER association (vertebral defects, anorectal atresia, tracheoesophageal fistula, renal anomalies)	Unknown (nosocomial)	32	21	Intestinal	ARDS, coagulopathy
4	Male	144	35	Cranial trauma	Unknown (nosocomial)	30	32	No	ARF, hyperkalemia, refractory intracranial hypertension
5	Male	36	16	No	Meningococcus	43	25	Four limbs (severe), cutaneous, intestinal	ARF, rabdomyolisis, severe metabolic acidosis
6	Male	15	12	No	Meningococcus	24	35	Limbs, cutaneous	No
7	Female	7	7	No	Meningococcus	23	19	Limbs, cutaneous	ARF, coagulopathy
8	Male	46	20	No	Unknown	12	26	No	Severe rabdomyolisis
9	Male	56	32	No	Meningococcus	23	30	No	Prior cardiac arrest
10	Female	2	4	Congenital metabolic disease?	Unknown	22	18	No	ARF, metabolic acidosis
11	Male	156	43	Cerebral palsy	Unknown (nosocomial)	31	19	No	No
12	Male	46	18	No	Meningococcus	23	24	No	Coagulopathy
13	Male	115	39	No	Unknown (pneumococcus?)	20	27	Limbs, cutaneous	Prior cardiac arrest, ARDS, ARF
14	Female	72	25	Rabdomyosarcoma	*Staphylococcus aureus*	52	43	No	No
15	Male	24	13	No	Meningococcus	33	22	Limbs, cutaneous	ARF, coagulopathy
16	Male	1	4	Propionic acidemia	*Staphylococcus aureus*	21	24	No	Severe metabolic acidosis

ARF, acute renal failure; ARDS, acute respiratory distress syndrome.

**Table 2:** Terlipressin (TP) treatment and outcome

Patient	Time from PICU admission to TP therapy (hours)	Time of maintenance of TP therapy (hours)	Adverse effects^a^	PICU length of stay (days)	Survival of episode	Cause of death	Sequela
1	40	48	No	49	Yes		Minor amputation (one right hand finger)
2	4	32	Ischemia (limited to toes)	7	Yes		No
3	12	65	No	51	No	Refractory shock	
4	6	3	Severe limbs and cutaneous ischemia, hyperkalemia	6	No	Ventricular fibrillation	
5	24	24	Limbs and cutaneous ischemia	22	Yes		Major amputation: lower limbs (below knees) and both hands
6	72	102	No	49	Yes		Minor amputation (one hand finger)
7	26	8	Severe limbs ischemia, cutaneous ischemia, hyperbilirubinemia	14	No	Withdraw of therapy	
8	48	20	Rabdomyolisis?	22	Yes		No
9	4	5	Oliguria	9	Yes		Partial anopsy, dismetry
10	32	96	Cutaneous and intestinal ischemia	6	No	Withdraw of therapy	
11	168	52	Limb and cutaneous ischemia, oliguria	8	No	Multiorgan failure	
12	14	7	No	1	No	Arrhythmia	
13	60	8	No	3	No	Ventricular fibrillation	
14	20	20	No	1	No	Refractory shock	
15	5	18	Limbs ischemia, rabdomyolisis	2	No	Refractory shock	
16	48	72	Limbs and cutaneous ischemia	14	Yes		

PICU, pediatric intensive care unit. ^a^Based in the opinion of the responsible physician

**Table 3:** Evolution of hemodynamic variables and catecholamine infusion rates after terlipressin therapy

	Before terlipressin therapy	Time after terlipressin therapy					
		
		30 min	60 min	4 hours	12 hours	24 hours	48 hours
Systolic blood pressure (mmHg)	77 (50–140)	108 (61–154)*	102.5 (61–137)*	99 (65–147)	91 (70–120)	107 (55–118)	105 (65–130)
Mean blood pressure (mmHg)	50.5 (37–93)	77 (42–100)**	69.5 (41–104)*	74 (40–95)	62 (40–90)	68 (35–90)	73 (40–103)
Diastolic blood pressure (mmHg)	38 (25–70)	57 (32–72)*	55.5 (31–90)*	48 (25–86)	50 (20–80)	48 (26–77)	53 (30–90)
Heart rate (beats/min)	155 (80–205)	149 (114–186)	148 (85–190)	148 (110–190)	146 (114–185)	142 (102–170)	148 (101–170)
Central venous pressure (mmHg)	14 (4–23)	13 (3–23)	12.5 (3–17)	13 (3–27)	12 (4–24)	12 (5–18)	13.6 (5–22)
Catecholamines (µg/kg/min)							
Noradrenaline	2 (1–4)	1 (0–3)	1.15 (0–3)	1.4 (0–2)	1 (0–2.6)*	1 (0–2.5)**	0.1 (0–1)**
Adrenaline	1.2 (0.4–4)	1 (0.5–6)	1 (0.3–4)	0.7 (0.2–3)	0.6 (0.1–2)	1 (0.2–2)	0.5 (0–2.5)
Dopamine	21.5 (10–52)	16.3 (3–40)	17.5 (0–52)	10 (0–40)	15.8 (0–40)	20 (3–40)	11.6 (0–45)
Dobutamine	22.5 (5–40)	20 (0–40)	20 (0–40)	20 (0–40)	20 (0–40)	20 (0–40)	22.5 (10–30)
Inotropic equivalent^a^	176 (141–552)		153.5 (67–460)		128.5 (57–340)	117.5 (20–340)*	82 (0–371)**

^a^Inotropic equivalent: (noradrenaline × 100) + (adrenaline × 100) + dopamine + dobutamine + (milrinone × 15) [25].

* *P *< 0.05 versus baseline. ** *P *< 0.01 versus baseline.
